# *Vairimorpha* (*Nosema*) *ceranae* Infection Alters Honey Bee Microbiota Composition and Sustains the Survival of Adult Honey Bees

**DOI:** 10.3390/biology10090905

**Published:** 2021-09-13

**Authors:** Yakun Zhang, Meiling Su, Long Wang, Shaokang Huang, Songkun Su, Wei-Fone Huang

**Affiliations:** 1 College of Animal Science (College of Bee Science), Fujian Agriculture and Forestry University, Fuzhou 350002, China; yakun.zhyk@gmail.com (Y.Z.); sumeiling1996@163.com (M.S.); wl1374286172@163.com (L.W.); skhuang@fafu.edu.cn (S.H.); susongkun@zju.edu.cn (S.S.); 2 Fujian Honey Bee Biology Observation Station, Ministry of Agriculture and Rural Affairs, Fuzhou 350002, China

**Keywords:** nosema disease, microsporidia, probiotics, prebiotics, microbiota, honeybees

## Abstract

**Simple Summary:**

The gut microbiota, in addition to the hosts and the pathogens, has become the third factor involved in gut disease developments, including honey bees. Interestingly, various studies reported positive associations between the gut bacteria and the most commonly found microsporidian pathogen instead of negative associations. To investigate the positive associations, a prebiotic that also exists in honey was added in the trials. Bees fed the prebiotics have slightly higher pathogen counts but lower mortalities. Microbiota analyses suggested that bees with the infection have a microbiota composition similar to that of bees with a longer lifespan, and the prebiotic seemed to enhance the similarities. Since microsporidia typically cause chronic infections, the positive associations may serve to sustain the host lifespans which is the optimal outcome for the pathogen that the survived bees can withstand pathogen proliferation and transmit the pathogens. Although the mechanisms underlying the associations were not revealed, this study indicated that nosema disease management in bees through changes in microbiota may shorten the lifespans or enhance both the infection and the bee population. Such results have appeared in recent field studies. More studies will be needed for the disease management using bee gut microbiota.

**Abstract:**

*Vairimorpha* (*Nosema*) *ceranae* is the most common eukaryotic gut pathogen in honey bees. Infection is typically chronic but may result in mortality. Gut microbiota is a factor that was recently noted for gut infectious disease development. Interestingly, studies identified positive, instead of negative, associations between core bacteria of honey bee microbiota and *V. ceranae* infection. To investigate the effects of the positive associations, we added isomaltooligosaccharide (IMO), a prebiotic sugar also found in honey, to enhance the positive associations, and we then investigated the infection and the gut microbiota alterations using qPCR and 16S rRNA gene sequencing. We found that infected bees fed IMO had significantly higher *V. ceranae* spore counts but lower mortalities. In microbiota comparisons, *V. ceranae* infections alone significantly enhanced the overall microbiota population in the honey bee hindgut and feces; all monitored core bacteria significantly increased in the quantities but not all in the population ratios. The microbiota alterations caused by the infection were enhanced with IMO, and these alterations were similar to the differences found in bees that naturally have longer lifespans. Although our results did not clarify the causations of the positive associations between the infections and microbiota, the associations seemed to sustain the host survival and benefit the pathogen. Enhancing indigenous gut microbe to control nosema disease may result in an increment of bee populations but not the control of the pathogen. This interaction between the pathogen and microbiota potentially enhances disease transmission and avoids the social immune responses that diseased bees die prematurely to curb the disease from spreading within colonies.

## 1. Introduction

Microsporidia are a group of intracellular eukaryotic microorganisms that are closely related to the Fungi. These naturally occurring pathogens often cause disease in laboratory animals that are reared in a dense population [1]. Honey bees are eusocial insects that intimately interact within densely populated colonies, creating a suitable environment for pathogens, including microsporidia. There are two common microsporidian species, *Varirmorpha* (*Nosema*) *apis* and *Vairimorpha* (*Nosema*) *ceranae* (genus recently redefined [2]) identified in European honey bees, *Apis mellifera*. *V. ceranae* is the most common gut pathogen in adult honey bees [3], causing a significant reduction of honey production, and subtle changes that may decrease colony fitness [4].

*Vairimorpha ceranae* was firstly identified in the Asian honey bee, *Apis cerana*, and is usually described as an emerging pathogen in *A. mellifera* [5]. Early studies suggested that no host barrier exists in *A. mellifera* for *V. ceranae* infection [6]; consequently, the earliest *V. ceranae* infection in *A. mellifera* was traced back to 1979 in Brazil [7]. Trials [8] have suggested that *V. ceranae* has a host range that includes most honey bee species. The pathogen may have adapted to the gut environment over the ensuing decades.

Chronic infections are commonly described for microsporidian species [9]. *V. ceranae* infections mainly occurred in midgut epithelium cells and have characteristics of typical chronic infections [10], including hormonal and immune responses [11,12] and subtle alterations of behavior [13]. Many studies suggest that *V. ceranae* significantly reduces longevity, but this effect is not universally reported [14,15]; differences among repeat trials made mortality insignificant in cages [16] and hives [17], which suggested unknown factors affect the mortality caused by the infection. In addition, feeding pollen, the major protein and polysaccharide source in the honey bee diet, increased the host longevity and the *V. ceranae* numbers [18,19]. Pollen feeding alters bee physiology, development, and gut microbiota [20].

Honey bee gut microbiota is simple and mainly consists of a small group of core bacterial species [21,22,23]. Although midgut microbiota transited according to the division work [24], the midgut harbors relatively few gut bacteria, possibly due to the constantly regenerated peritrophic membrane [25]. The hindgut has a narrowed ileum with complicated structures in cross-section and a balloon-like rectum for storing feces. Similar to most animals, the honey bee ileum and rectum harbor abundant gut microbes [22,25]. Among the core bacteria, *Lactobacillus* Firm-4 and Firm-5, *Bifidobacterium* spp., *Gilliamella apicola*, and *Snodgrassella alvi*, dominate the hindgut environment. These bacteria affect various physiological functions of bees [21,22] and are recognized as necessary to maintain homeostasis and as beneficial symbionts in the gut environment.

Independent studies have shown positive associations between *V. ceranae* infections and hindgut bacteria populations. *G. apicola* was significantly and positively associated with *V. ceranae* infections [26]. A positive association of *S. alvi* and *V. ceranae* infection was identified in *A. cerana* [27], and *Bifidobacteria* spp. in *A. mellifera* [28]. The positive associations of bifidobacteria and *G. apicola* were recently confirmed in another independent study [29], and microsporidian infection was positively associated with *Snodgrassella* spp. in bumble bees [30]. These studies suggested positive associations between the microsporidian pathogen and the gut core bacteria species. Such positive associations seem counterintuitive since some associated bacteria are generally believed to be probiotics that possibly inhibit pathogenic bacteria [31]. Although most studies of microbiota/pathogen in animals aim to identify any associations, agonistic associations seem more appealing due to the possible applications in treatments. Positive associations, however, suggested the opposite. Nevertheless, these microbes that provide benefits to the host may not contradict the needs of a pathogen. Chronic pathogens, i.e., microsporidia, rely on the infected host to tolerate the infection and remain active during the time the pathogen reproduces and disseminates. Therefore, we hypothesized that *V. ceranae* may have adapted to the gut microbiota and modulated them to maintain host homeostasis, leading to tolerance of the infection and high prevalence of the disease.

In this study, we used a prebiotic to interfere with the associations and evaluate our hypothesis of the positive associations between *V. ceranae* and honey bee gut bacteria. Isomaltooligosaccharide (IMO) was selected as the prebiotic because isomaltose (the disaccharide form of IMO) is a common sugar found in honey, and it can enhance lactobacilli and bifidobacteria [32,33]. Results of the caged bees indicated that infected bees feeding IMO have significantly higher *V. ceranae* spore counts but significantly lower mortality. The infection alone has doubled the hindgut bacteria quantities, and IMO resulted in specific microbiota alterations. In addition to the previously identified positive associations, we found that *Commensalibacter* and *Bartonella* populations were also positively associated with the infection in the fecal microbiome. These gut microbiota alterations were coincident with microbiome comparison for summer and winter foragers [34]; the latter can survive much longer. Therefore, our hypothesis that these positive associations serve to sustain the host survival, a benefit for both gut bacteria and *V. ceranae*, is supported by the results. Moreover, sustaining host survival may prevent an apoptosis-like social immune response in which infected bees die prematurely to stop pathogen transmission within the colony.

## 2. Materials and Methods

### 2.1. Honey Bee Rearing and Inoculation

Honey bee colonies, commercially available hybrids with phenotypes of *Apis mellifera ligustica*, were locally purchased from an apiary in Fuzhou. The rearing method and conditions were identical to those of our previous work [28,35]. Briefly, newly emerged workers, within 24 h, were collected from three brood frames that were pulled from three different randomly selected colonies and kept in a 34 °C growth chamber. The newly emerged bees freely ingested nectar and bee bread stored on the brood frame before the collection. The collected bees were transferred into round food-grade polypropylene containers (480 mL) with artificial pollen patties and a gravity feeder. Six cages, 100 bees per cage, were prepared and randomly selected for one of two sugar-water types, IMO and sucrose. The IMO solution contained 10% isomaltooligosaccharide (IMO), 45% sucrose, and 45% ddH_2_O, and the sucrose solution contained 50% sucrose and 50% ddH2O, all in *wt/wt*. IMO, purchased from Yuanye Bio (Shanghai, China), was food-grade fermented from starches and mainly resulted in disaccharides with the degree of polymerization up to five [32]. We decided to use 10% IMO to replace 5% sucrose because of the potential indigestible nature of IMO, and the sugar–water consumptions were recorded in the trials to evaluate if this ratio difference alters sugar consumption. Artificial pollen patties and the sugar water solutions were fed ad libitum. The bees were held in a 34 °C growth chamber for five days until inoculation.

*Vairimorpha ceranae* spores were freshly isolated from foragers collected on the campus apiary, and only *V. ceranae* was identified [28]. Spores were isolated using the same method reported in our previous work [28,35]. In brief, approximately 20 returning foragers with pollen were collected at hive entrances, and the midguts were pulled out from abdomens. Pooled midguts were macerated in PBS and centrifuged at 6000× *g* for five minutes. Supernatants and tissue debris were removed carefully without disturbing the precipitated spores. The spores were resuspended in 1 mL PBS and then centrifuged again; supernatant and any visible tissue debris were removed, and this washing process was repeated twice. Purified spores were counted in a hemocytometer and used immediately to inoculate bees. The inoculation method used in previously published studies was applied [28,35]. Briefly, five-day-old bees from the same cage were randomly divided into treated and control groups after anesthesia on ice. Bees were fastened individually on a Styrofoam board, and the 2 μL sugar water (10^5^ spores for the treated group, and only sugar water for the control) was given to the bees upon awake. At 30 min post-feeding, bees that completely ingested the sugar-water were transferred to new cages, 45–50 bees per cage, and given the same sugar-water type and pollen patties that they were fed before inoculation. Twelve cages were generated, three cages each for bees inoculated with *V. ceranae* and fed either IMO or sucrose and two control groups of three cages each for bees fed IMO or sucrose without inoculation. The cages were held in a 30 °C growth chamber, 24 h dark, 70% relative humidity.

### 2.2. Examination of Caged Bees

The cages were checked daily after inoculation. The dead bees were removed and recorded for survival analysis (the Kaplan–Meier method using SPSS23, IBM). Sugar–water consumption was recorded every 3 d when the feeders were replaced to monitor if the sugar-water types affect the consumptions.

To evaluate the infection intensity and bacterial microbiota, five bees were randomly collected from the cages at 12 d post-inoculation (dpi) and then every other day. Collected bees were anesthesia on ice and then dissected on a clean dish plate. Abdominal cuticles were carefully torn away using tweezers to obtain intact alimentary organs. The midgut, the hindgut, and the feces were separated for individual storage. First, the midgut was separated from the hindgut by a cut at the junction where Malpighian tubes attach. The dissected midgut was stored in 100 μL PBS and macerated for microscopic examination later. The hindgut with the balloon-like rectum, which contains accumulated feces of the entire caged period, was transferred into another tube containing 50 μL TE buffer, and then the rectum was broken in the TE buffer using a clean tweezer to collect the feces. The hindgut sample, including the ileum and the broken rectum, was washed twice in TE buffer to remove fecal residues before storing them individually in 50 μL TE buffer. The infected bees with midgut spore count <10^7^, the approximate plateau phase level of *V. ceranae* infection [35,36] were not included in the following qPCR and 16S rRNA gene sequencing.

### 2.3. DNA Extraction and Quantification of Bacteria of Hindguts and Feces

DNA of hindgut and feces was extracted by the Chelex method [35]; briefly, 5 μL of homogenized sample transferred into 55 μL Chelex buffer (10% Chelex 100 resin, Bio-rad, 1% Triton X-100; in *wt/wt*) and vortex at the strongest setting for 15 sec, and then transferred the mixture into a 200 μL PCR tube with 3 μL proteinase K (20 mg/mL). The tubes were incubated (56 °C for two hrs, 95 °C for 30 min, and then 4 °C for 30 s) in a CFX connect (Bio-rad, Hercules, CA, USA) thermocycler, and then centrifuged at 14,000× *g* for 10 min. The supernatants were used as DNA solutions in the following qPCR. The qPCR methods were identical with our previous work [28]; in brief, ChamQ universal SYBR qPCR mixture (Vazyme, Nanjing, China) and CFX-384 (Bio-rad, Hercules, CA, USA) were used. Primers for quantifying the core bacteria were published in previous honey bee gut microbiota studies [37,38], and the sequences are listed in Supplementary Table S1. Honey bee actin primer set [39] and the universal qPCR primer set for all bacteria [40] was used to normalize the bacteria qPCR results. All qPCR conditions (the reaction temperatures and the primer concentrations were listed in Table S1) were adjusted according to the primer set to make sure that all qPCR have similar PCR efficiencies within the acceptable range. The significance of the differences in results was calculated using SPSS 23 (IBM). All data sets were tested for normality using the Kolmogorov–Smirnov method. The dataset that passed the normality test was compared and significance was found by one-way or two-way ANOVA. The Kruskal–Wallis method was applied to the dataset that did not pass the normality test.

### 2.4. Fecal Microbiome Comparisons

Fecal samples were submitted for 16S rRNA gene sequencing using Illumina Miseq because we noted that qPCR may not generate robust results from fecal samples. DNA samples from six bees of the same group were pooled and five pooled fecal samples of each group were submitted for sequencing. The samples were PCR amplified using a universal primer set, 338f/806r, targeting the V3–V4 region, with adaptor sequence for the Miseq library and then sequenced on Miseq (Illumina) with the assistance of Majorbio (Shanghai, China). Briefly, the PCR was performed using Fastpfu DNA polymerase (TransGen, Beijing, China) in 30 cycles. PCR products were revealed in 2% gel-electrophoresis, and products in the expected size range were extracted using AxyPrepDNA kit (Axygen) and quantified using QuantiFluor™ (Promega). The PCR products were then processed using TruSeqTM DNA Sample Prep Kit (Illumina) before sequencing. The obtained data were processed using preset settings and analyzed on the Majorbio Cloud Platform (www.majorbio.com (accessed on 4 November 2019)). Briefly, the single reads were excluded from the results, and the operational taxonomic units (OTU) that shared more than 97% identity were recognized as the same species. Silva database (Release132, http://www.arb-silva.de; accessed on 4 November 2019) was used for the species annotations.

## 3. Results

### 3.1. Sugar Consumption and Mortality of the Caged Bees

**Figure 1:**
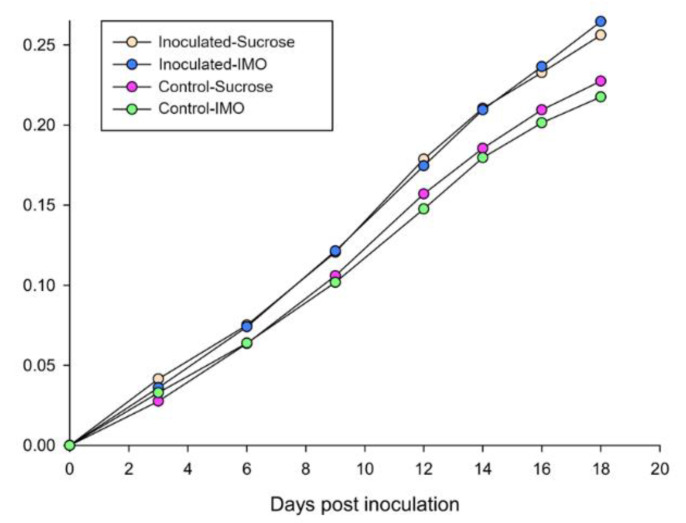
Accumulated sugar water consumption of the four honey bee treatment groups. Y-axis indicates the accumulated sugar–water consumption in grams. The four treatment groups were generated by two variables: IMO/sucrose and *V. ceranae* inoculation/control.

**Figure 2:**
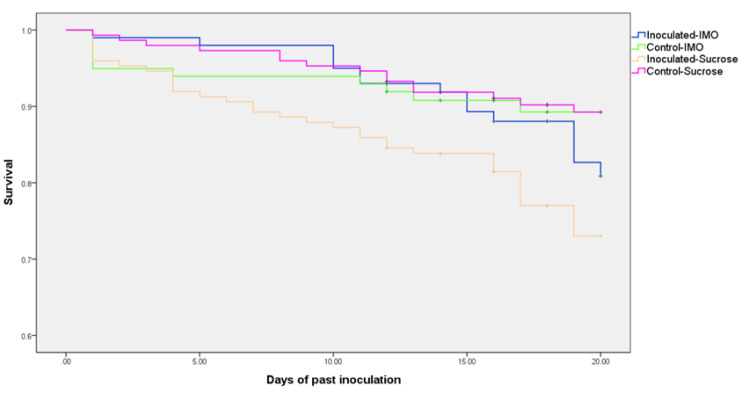
Survival analysis using the Kaplan–Meier method of the four treatment groups. Only the group inoculated with sucrose showed a significant increase in mortality.

No significant difference in consumption of sugar water was found between IMO and sucrose groups, which suggested that IMO and the sugar ratios did not cause significant alterations. As expected, *V. ceranae*-inoculated groups consumed significantly more sugar water, both IMO and sucrose groups, than the uninfected controls (*p* = 0.034, Figure 1). There was no difference in mortality between control groups fed the two types of sugar waters; however, mortality was significantly lower (*p* < 0.001) in the infected group fed IMO compared to the infected group fed only sucrose (Figure 2). Mortality was not different for *V. ceranae*-inoculated bees fed IMO and uninfected control bees fed either solution.

### 3.2. Differences in V. ceranae Infection Intensities

**Figure 3:**
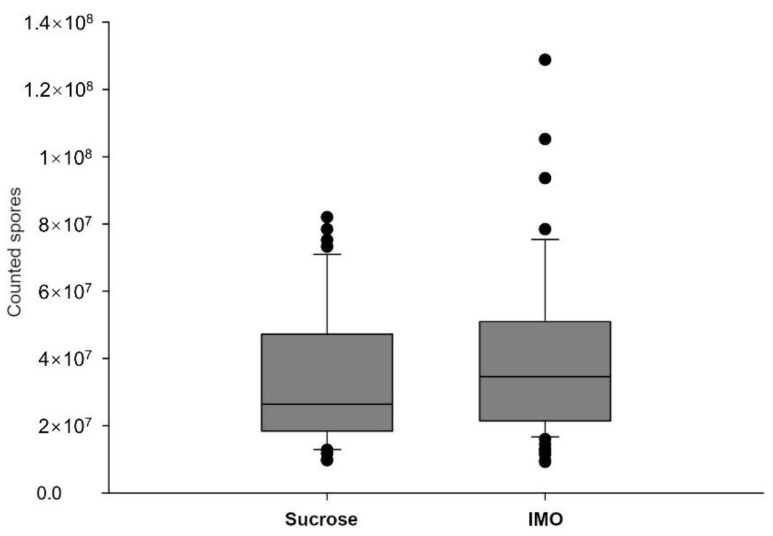
Infection intensities of the inoculated groups fed sucrose and IMO sugar–water. Each bee midgut was individually processed and spores counted under a phase-contrast microscope. Control bees were free of visible spores.

To evaluate infection intensities in the midgut, five bees were analyzed individually by microscopy from each cage at 12 dpi and then every other day. The trial was terminated at 20 dpi because fewer than five bees survived in the cages. The inoculation resulted in a 100% infection rate in the collected bees, and the uninoculated bees were free of spores. Infected midguts had slightly to substantially enlarged sizes, but the ileum and the rectum parts have no visible differences between infected and uninfected groups. No visible difference of the dissected midguts and hindguts was noted between IMO and sucrose groups. Midguts of the same group had similar infection intensities collected in 12–20 dpi, reaching the plateau phase. The *V. ceranae* spore counts were slightly, but significantly (*p* = 0.032, the Kruskal–Wallis method), higher in IMO groups (Figure 3). Individual differences and variations were found among the samples, and the bees fed IMO tended to have higher spore counts in the dissected midguts. Because we were investigating fully developed infections, infected bees with less than 10^7^ spores were not included in the following analyses.

### 3.3. Quantification PCR of Core Bacteria

**Figure 4:**
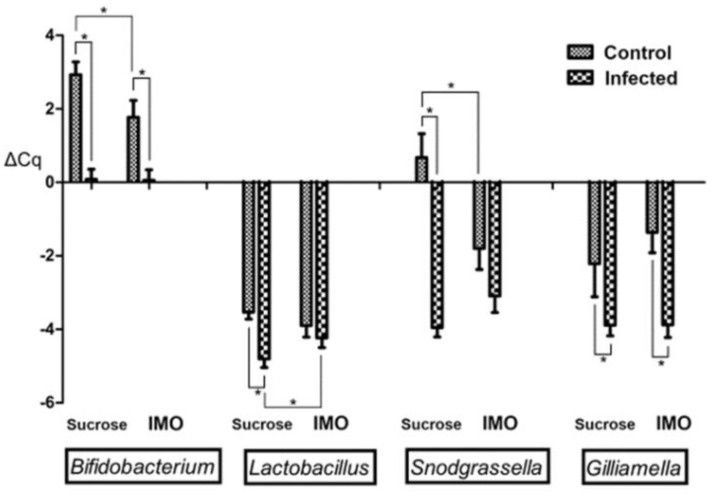
Relative qPCR results of the hindgut samples normalized using honey bee Beta-actin. The lower ΔCq values indicate higher bacteria populations. Significances are labeled by asterisks.

**Figure 5:**
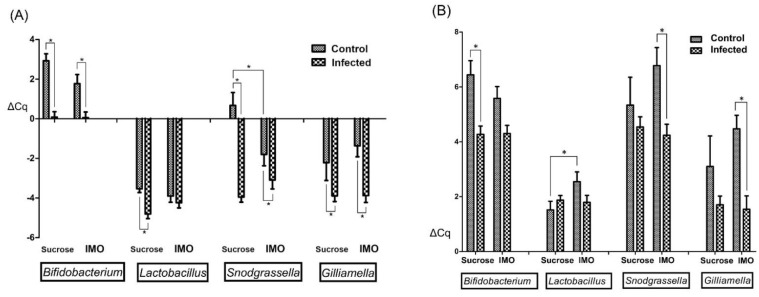
Relative qPCR results of the hindgut and fecal samples indicating the population ratio alterations. The results were normalized by the qPCR results of universal bacteria primers. The lower ΔCq indicates the higher ratio within the gut microbiota. (**A**) Hindgut results, (**B**) Fecal sample qPCR results. Significances are labeled by asterisks.

Four core-bacteria species with higher populations in the microbiota, *Lactobacillus* spp., *Bifidobacterium* spp., *Snodgrassella alvi*, and *Gilliamella apicola*, were included in the qPCR. To conduct a relative quantification, we attempted to normalize the results using both the honey bee Beta-actin gene and the universal bacteria primer sets, but the Beta-actin was not able to serve as the reference in fecal samples because of low (>35) or no Ct values. Although the universal bacteria primer set yielded reliable Ct values in both hindgut and fecal samples, surprisingly, the Ct values were significantly lower (*p* < 0.001, the Kruskal-Wallis method) in the *V. ceranae* infected samples, both hindgut (mean Ct = 21.33 ± 0.148 in infected groups vs. 22.34 ± 0.305 in uninfected groups) and fecal samples (mean Ct = 20.71 ± 0.244 vs. 23.53 ± 0.210). Interestingly, IMO feeding did not significantly (*p* = 0.897 in hindgut, and *p* = 0.337 in fecal samples) alter the values of the universal bacteria primers results, as expected. This observation was confirmed using the Beta-actin results to normalize the universal bacteria qPCR results of hindgut samples. Infected bees have more than doubled bacterial population (ΔΔCq = 1.45, infected bees have mean ΔCt = −4.70 ± 0.153 and un-infected bees have ΔCt = −3.25 ± 0.184) in the hindgut samples that included ileum and rectum linings; IMO feeding resulted in ΔΔCq value of 0.21, which is smaller than the difference that qPCR can reliably distinguish. Although the overall microbiota was altered by the infection, the qPCR results normalized using the universal bacteria primers can indicate the population ratio alterations within the microbiota, which is similar to the population analysis commonly included in microbiome studies using 16S rRNA gene sequencing. Therefore, we decided to keep the hindgut results normalized using Beta-actin (Figure 4) to show the relative quantity alterations, and the hindgut and the fecal samples normalized using the universal bacterial primers that can indicate population ratio alterations were presented in Figure 5A,B. Before comparing the means and significance in the figures, we noted that not all data passed the Kolmogorov–Smirnov exam of normality; many groups showed significance (*p* < 0.05) or at the marginal value (*p* = 0.20) in the normality exam. Therefore, the Kruskal–Wallis method was applied to find the significance between the groups.

*Vairimorpha ceranae* infection and IMO feeding caused significant bacteria alterations in the hindgut (Figure 4). *V. ceranae* infection significantly increased all monitored bacteria (*p* < 0.01) in the groups feeding only sucrose; however, only bifidobacteria (*p* = 0.009) and *G. apicola* (*p* < 0.001) were significantly altered in the IMO feeding groups, possibly caused by the IMO feeding effects. IMO feeding significantly enhanced *S. alvi* (*p* = 0.020) in addition to bifidobacteria (*p* = 0.043) in the IMO feeding un-infected bees. Surprisingly, IMO feeding resulted in the opposite alterations in the *Lactobacillus* spp. quantities; IMO slightly but not significantly (*p* = 0.240) increased *Lactobacillus* spp. quantities in un-infected groups, but IMO significantly (*p* = 0.043) decreased Lactobacillus spp. in infected groups (Figure 4). Similar trends were noted in the hindgut bacteria ratio results that normalized using the universal bacteria primers results (Figure 5A). Infection increased all quantified core bacteria, except Lactobacillus spp., and IMO feeding enhanced the decline of *Lactobacillus* spp. in infected bees.

Fecal samples showed somehow different trends. Only the bifidobacteria were significantly increased (*p* < 0.001) in infected bees fed only sucrose. In infected bees feeding IMO, the ratios of all the four core bacteria were significantly (*p* < 0.05) increased. The lactobacilli ratio was significantly decreased in uninfected bees feeding IMO. In addition, we noted that not all fecal samples generated detectable Ct values of *S. alvi* and *G. apicola*, and the sample numbers of the *G. apicola* qPCR results were lower than the number that can provide a meaningful statistical result in comparisons. The correlation analysis suggested that the presence of qPCR detectable *S. alvi* and *G. apicola* in fecal samples were significantly correlated with the *V. ceranae* infection (*p* < 0.001). All the data presented in the figures were listed in Table S2 in mean ± SEM with the sample number of each group.

### 3.4. 16S rRNA Gene Sequencing of Fecal Samples

**Figure 6:**
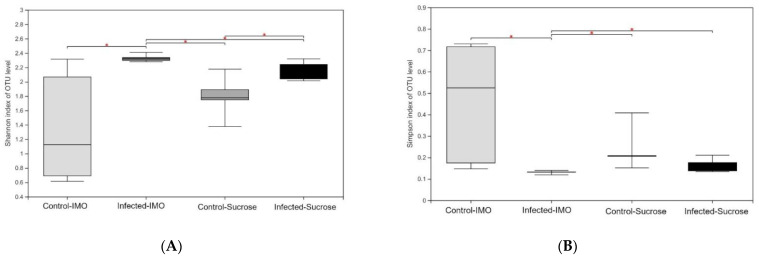
Alpha diversity analyses. (**A**) Shannon index. (**B**) Simpson index. The infected group receiving IMO had a significantly higher Shannon index value (2.329, *p* = 0.012) and lower Simpson index value (0.130, *p* = 0.012). Significances (*p* < 0.05) are labeled by asterisks.

The sequencing results of the fecal samples, 962,214 sequences in total, and the average length was 424 bp, showed significant differences. In the alpha diversity analyses (Figure 6), the infected groups showed higher Shannon index values and lower Simpson index values, which suggested that *V. ceranae* infection significantly (*p* < 0.05) reduces the alpha diversity of the fecal microbiota. In addition, the infected group fed IMO showed the most distinctive changes in the analyses (Figure 6). Feeding IMO alone did not result in any significant alteration in the microbiota diversity (*p* > 0.05, alpha diversity analyses), but the within-group differences were much higher than those in other groups (Figure 6). Infected bees fed IMO showed the opposite results, the lowest within-group differences. Beta diversity analyses suggested that all the infected samples were grouped in hierarchical clustering and Principal Co-ordinates Analysis (PCoA). In permutational multivariate analysis of variance (PERMANOVA) with Bray–Curtis method with 1000 times of replicates, *V. ceranae* infection alone significantly affected the microbiota composition (*p* = 0.002), and IMO feeding alone did not (*p* = 0.488).

**Figure 7:**
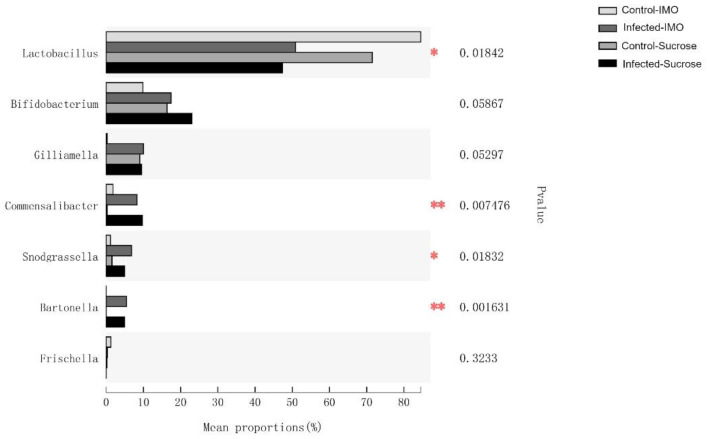
The differences in bacterial population ratios among the treatment groups. The multiple group comparisons were calculated using the Kruskal–Wallis test with the False Discovery Rate (FDR) approach and Welch’s posthoc method. The comparisons were made at the bacterial genus level. Significances (*p* < 0.05) are labeled by asterisks, and highly significances (*p* < 0.01) are labeled by double asterisks.

Bacterial population ratio analysis at the genus level indicated that *Lactobacillus*, *Commensalibacter*, *Snodgrassella*, and *Bartonella* populations were significantly different among the groups; *Bifidobacterium* and *Gilliamella* were slightly different (Figure 7, using the Kruskal–Wallis method). Comparing the infected and control groups, all the core bacteria species were significantly altered. Neither was there a significant alteration in bacterial population ratios between control groups fed sucrose and IMO, although the mean value was different (Figure 7), possibly because of the high diversity and deviation of the control group fed IMO, shown in Figure 6.

## 4. Discussion

Positive associations between the microsporidian, *V. ceranae*, and several core bacteria symbionts in bees, including *Bifidobacterium* spp. [28], *Snodgrassella alvi* [27,30], and *Gilliamella apicola* [26], led to the hypothesis that these enhanced bacteria may positively affect host homeostasis that possibly led to the tolerance and the mortality discrepancies in studies. Foragers can carry millions of spores without obvious symptoms, and the mortality discrepancies among studies using different subspecies of honey bees, foods, and environmental settings [14] although no specific trend was noted in studies. Many associated core bacteria are considered probiotics in other animals and are expected to add health benefits, including protection from pathogens [41,42] and additional lifespan [43]. In this study, we found IMO feeding helped the infected bees survive as well as the control bees in identical settings, and the spore load was slightly increased. We then investigated the changes in the gut microbiota and found that IMO feeding resulted in an unexpected decrease of *Lactobacillus* spp., especially in infected bees. *Lactobacillus* spp. have the capabilities to digest IMO and increase the population [32], but feeding IMO significantly decreased the lactobacilli quantities in infected bees (Figure 4) although infected bees ingested more IMO sugar–water (Figure 1). This effect was even more prominent in the population ratio (Figure 5A,B). Although *Lactobacillus* spp. has no known specific physiological function in the bees [21], *Lactobacillus* spp. and the metabolites could reduce *V. ceranae* infection intensity [44]. The decrease in *Lactobacillus* spp. may cause spore count increment in the IMO feeding bees.

Associations identified between the microbiota and the host longevity may explain the survivorship enhancement of IMO feeding bees. The numbers of *Bifidobacterium* and *Snodgrassella* spp. in the rectum were positively associated with longevity in the comparison of worker and queen bees [45], and these two groups of bacteria were both increased by the IMO feeding and the infection in our results. The increase of overall microbiota quantities, the ratios of *Commensalibacter* and *Bartonella* population, and the reduced microbiome alpha diversity in the *V. ceranae*-infected bees coincided with the results found in the comparisons between summer and winter foragers [34]; the latter had longer lifespans. Therefore, our hypothesis that the associations between *V. ceranae* and microbiota have positive effects should be correct.

*Vairimorpha ceranae* infection may modulate the host’s core bacteria by affecting polysaccharide digestion, in addition to the hypothesized immune modulations [46]. We found the pronounced IMO effects of decreasing *Lactobacillus* spp. only in the *V. ceranae* infected bees, which is the opposite of IMO in vitro effects [33]. The isomaltooligosaccharides (IMO) we used are mostly small oligos that lactobacilli may utilize, and honey bees also have enzymes to digest such small oligos [47]. *V. ceranae* infection may have impaired or altered polysaccharide digestion; however, the expressions of related genes were upregulated in transcriptome [48] but differentially regulated in proteomic profiles [49]. Interestingly, fumagillin, the antibiotic treatment for *Nosema* disease in bees, also affected the modification of polysaccharide digestion enzymes and enhanced the infection intensity of *V. ceranae* at low levels [50]. However, the mechanisms of how digestion and infection affect bee microbiota remain unrevealed.

Similar results to our IMO feeding trial were reported in the investigations of feeding pollen—infection intensity increased [18,51], and mortality was reduced [18]; however, the possible microbiota alterations were not analyzed in these studies. Because pollen is the main polysaccharide source in the honey bee diet, we speculated that pollen feeding and *V. ceranae* infections altered the bee microbiota in those studies. Similar results were obtained when indigenous gut bacteria were gavaged into bees with microsporidian infection [52]—the infected and gavaged bees survived significantly longer than the bees without gavage. Feeding antibiotics and *V. ceranae* to bees showed the opposite results—mortality increased [53]. Overall, these independent studies generated similar results that support the concept that an enhanced indigenous gut microbiota population in bees may reduce the mortality caused by *V. ceranae* and increase the spore loads. However, only live bees were analyzed in these studies, including this study, and there is no evidence to suggest that bees dying prematurely from the infection have unaltered microbiota. In addition, one study suggested that modulations and positive associations may not exist in the early infection stages [54].

The results of our study suggest a potentially mutualistic interaction exists between honey bee gut microbiota and *V. ceranae*. Adding pollen and polysaccharides that bees can naturally ingest to the caged bees seemed to allow *V. ceranae* and microbiota to re-establish the interactions and reduce mortalities. In infected hosts receiving IMO polysaccharides, the populations of both *V. ceranae* and the associated bacteria were higher, and the host lifespan was not affected in this study. Studies feeding pollen [18] or bacteria [52] showed similar mortality reduction effects. Moreover, sustaining the host lifespan could be a strategy to avoid social immune reactions of the honey bee colony as a super-organism [55,56]. A social immune response includes the premature death of infected individuals to stop the spread of disease, similar to apoptosis by infected or damaged cells. Because social immune response in honey bees may be efficient enough to drastically reduce immune-related genes in the honey bee genome [57], it could also be the selection force that leads to the associations between *V. ceranae* and gut bacteria.

*Vairimorpha ceranae* is not the first microsporidium that can affect gut bacteria [58], and microsporidia are not the only pathogens that can destroy or affect the host-microbiota [59]. Although there is no causation demonstrated in this study, we found the story of the interactions between honey bee gut microbiota and *V. ceranae* infections may not be straightforward. In addition, the interactions cannot be ignored in the pursuit of an integrated solution for controlling nosema disease. Since the interest in applying microbiota alterations to control honey bee diseases is growing [60,61], understanding the mechanisms underlying the interactions will be critical before large-scale field applications.

## 5. Conclusions

Honey bee gut microbiota has become an essential factor in the discussions for gut pathogens. The interactions among bees, gut microbiota, and gut pathogens are intriguing, in addition to the possible application in integrated disease management in honey bees. Nosema disease—the most common gut disease, which causes economical losses, possibly deteriorates the capabilities of bees against environmental stressors [4], and lacks effective treatments [50,60]—has incentivized multiple studies to investigate the possibilities of using dietary supplements [62], prebiotics [61,63,64], and gut bacteria, including indigenous [52], exogenous [65,66], and genetic modified [67], to control the disease. The reported nosema disease studies, however, suggest less conventional interactions, dissimilar to what has been found in bacterial diseases, e.g., adding symbiotic or harmless bacteria in the food and the environment can assist bees against foulbrood diseases [68,69]. This study demonstrated that enhancing indigenous gut microbiota may decrease mortality but not control the pathogen, and somewhat similar results have been reported in a field trial [61]. In addition to nosema disease, other non-bacterial gut pathogens, i.e., trypanosomatids and DWV, also reported somewhat counter-intuitive results [38,70]. These pathogens seemed not affected by adding indigenous gut microbes, and the host mortalities were decreased. These gut pathogens have adapted and co-evolved with the host microbiota since the origin of the diseases, far before the microbiota emerged as an attractive topic in pathogen studies. Therefore, the best outcome of the interactions among the host, the indigenous microbiota, and the pathogen may not be far from what we have seen in the field. Any changes that attempt to control the pathogens using microbes need to consider the possibilities of unintended deleterious effects to the bees. Further research to reveal the mechanisms underlying the interactions is needed.

## Data Availability

Not applicable.

## Supplementary Materials

The following are available online at https://www.mdpi.com/article/10.3390/biology10090905/s1. Table S1: The primers used in this study. Table S2: Data presented in Figure 4.

## References

[1] Moretto M.M., Khan I.A., Weiss L.M. (2012). Gastrointestinal Cell Mediated Immunity and the Microsporidia. PLoS Pathog..

[2] Tokarev Y.S., Huang W.-F., Solter L.F., Malysh J.M., Becnel J.J., Vossbrinck C.R. (2020). A formal redefinition of the genera *Nosema* and *Vairimorpha* (Microsporidia: Nosematidae) and reassignment of species based on molecular phylogenetics. J. Invertebr. Pathol..

[3] Chen Y., Evans J., Zhou L., Boncristiani H., Kimura K., Xiao T., Litkowski A., Pettis J.S. (2009). Asymmetrical coexistence of *Nosema ceranae* and *Nosema apis* in honey bees. J. Invertebr. Pathol..

[4] Botías C., Martín-Hernández R., Barrios L., Meana A., Higes M. (2013). *Nosema* spp. infection and its negative effects on honey bees (*Apis mellifera iberiensis*) at the colony level. Vet. Res..

[5] Higes M., Martín-Hernández R., Botías C., Garrido-Bailón E., González-Porto A.V., Barrios L., del Nozal M.J., Bernal J.L., Jiménez J.J., García-Palencia P. (2008). How natural infection by *Nosema ceranae* causes honeybee colony collapse. Environ. Microbiol..

[6] Huang W.-F., Jiang J.-H., Chen Y.-W., Wang C.-H. (2007). A *Nosema ceranae* isolate from the honeybee *Apis mellifera*. Apidologie.

[7] Teixeira E.W., dos Santos L.G., Sattler A., Message D., Alves M.L.T.M.F., Martins M., Grassi-Sella M.L., Francoy T.M. (2013). *Nosema ceranae* has been present in Brazil for more than three decades infecting Africanized honey bees. J. Invertebr. Pathol..

[8] Chaimanee V., Warrit N., Chantawannakul P. (2010). Infections of *Nosema ceranae* in four different honeybee species. J. Invertebr. Pathol..

[9] Solter L.F., Weiss L.M., Becnel J.J. (2014). Epizootiology of Microsporidiosis in Invertebrate Hosts. Microsporidia: Pathogens of Opportunity.

[10] Holt H.L., Aronstein K.A., Grozinger C.M. (2013). Chronic parasitization by *Nosema microsporidia* causes global expression changes in core nutritional, metabolic and behavioral pathways in honey bee workers (*Apis mellifera*). BMC Genom..

[11] Antúnez K., Martín-Hernández R., Prieto L., Meana A., Zunino P., Higes M. (2009). Immune suppression in the honey bee (*Apis mellifera*) following infection by *Nosema ceranae* (Microsporidia). Environ. Microbiol..

[12] Badaoui B., Fougeroux A., Petit F., Anselmo A., Gorni C., Cucurachi M., Cersini A., Granato A., Cardeti G., Formato G. (2017). RNA-sequence analysis of gene expression from honeybees (*Apis mellifera*) infected with *Nosema ceranae*. PLoS ONE.

[13] Wolf S., McMahon D.P., Lim K.S., Pull C.D., Clark S.J., Paxton R.J., Osborne J.L. (2014). So Near and Yet So Far: Harmonic Radar Reveals Reduced Homing Ability of Nosema Infected Honeybees. PLoS ONE.

[14] Fries I. (2010). *Nosema ceranae* in European honey bees (*Apis mellifera*). J. Invertebr. Pathol..

[15] Higes M., García-Palencia P., Hernández R.M., Meana A. (2007). Experimental infection of *Apis mellifera* honeybees with *Nosema ceranae* (Microsporidia). J. Invertebr. Pathol..

[16] Huang W.-F., Solter L., Aronstein K., Huang Z. (2015). Infectivity and virulence of *Nosema ceranae* and *Nosema apis* in commercially available North American honey bees. J. Invertebr. Pathol..

[17] Milbrath M.O., van Tran T., Huang W.-F., Solter L.F., Tarpy D., Lawrence F., Huang Z.Y. (2015). Comparative virulence and competition between *Nosema apis* and *Nosema ceranae* in honey bees (*Apis mellifera*). J. Invertebr. Pathol..

[18] Jack C.J., Uppala S.S., Lucas H.M., Sagili R.R. (2016). Effects of pollen dilution on infection of *Nosema ceranae* in honey bees. J. Insect Physiol..

[19] Zheng H.-Q., Lin Z.-G., Huang S.-K., Sohr A., Wu L., Chen Y.P. (2014). Spore Loads May Not be Used Alone as a Direct Indicator of the Severity of *Nosema ceranae* Infection in Honey Bees *Apis mellifera* (Hymenoptera:Apidae). J. Econ. Entomol..

[20] Ricigliano V.A., Fitz W., Copeland D.C., Mott B.M., Maes P., Floyd A.S., Dockstader A., Anderson K.E. (2017). The impact of pollen consumption on honey bee (*Apis mellifera*) digestive physiology and carbohydrate metabolism. Arch. Insect Biochem. Physiol..

[21] Kešnerová L., Mars R.A.T., Ellegaard K.M., Troilo M., Sauer U., Engel P. (2017). Disentangling metabolic functions of bacteria in the honey bee gut. PLoS Biol..

[22] Zheng H., Steele M.I., Leonard S.P., Motta E.V.S., Moran N.A. (2018). Honey bees as models for gut microbiota research. Lab Anim..

[23] Martinson V.G., Danforth B., Minckley R.L., Rueppell O., Tingek S., Moran N.A. (2011). A simple and distinctive microbiota associated with honey bees and bumble bees. Mol. Ecol..

[24] Cilia G., Fratini F., Tafi E., Mancini S., Turchi B., Sagona S., Cerri D., Felicioli A., Nanetti A. (2021). Changes of Western honey bee *Apis mellifera ligustica* (Spinola, 1806) ventriculus microbial profile related to their in-hive tasks. J. Apic. Res..

[25] Kwong W.K., Moran N.A. (2016). Gut microbial communities of social bees. Nat. Rev. Microbiol..

[26] Rubanov A., Russell K.A., Rothman J.A., Nieh J.C., McFrederick Q.S. (2019). Intensity of *Nosema ceranae* infection is associated with specific honey bee gut bacteria and weakly associated with gut microbiome structure. Sci. Rep..

[27] Huang S.K., Ye K.T., Huang W.F., Ying B.H., Su X., Lin L.H., Li J.H., Chen Y.P., Li J.L., Bao X.L. (2018). Influence of Feeding Type and *Nosema ceranae* Infection on the Gut Microbiota of *Apis cerana* Workers. mSystems.

[28] Zhang Y., Lu X., Huang S., Zhang L., Su S., Huang W.-F. (2019). *Nosema ceranae* infection enhances *Bifidobacterium* spp. abundances in the honey bee hindgut. Apidologie.

[29] Castelli L., Branchiccela B., Garrido M., Invernizzi C., Porrini M., Romero H., Santos E., Zunino P., Antúnez K. (2020). Impact of Nutritional Stress on Honeybee Gut Microbiota, Immunity, and *Nosema ceranae* Infection. Microb. Ecol..

[30] Cariveau D.P., Powell J., Koch H., Winfree R., Moran N.A. (2014). Variation in gut microbial communities and its association with pathogen infection in wild bumble bees (*Bombus*). ISME J..

[31] Mattila H.R., Rios D., Walker-Sperling V.E., Roeselers G., Newton I.L.G. (2012). Characterization of the Active Microbiotas Associated with Honey Bees Reveals Healthier and Broader Communities when Colonies are Genetically Diverse. PLoS ONE.

[32] Gänzle M.G., Follador R. (2012). Metabolism of Oligosaccharides and Starch in Lactobacilli: A Review. Front. Microbiol..

[33] Hu Y., Ketabi A., Buchko A., Gänzle M. (2013). Metabolism of isomalto-oligosaccharides by *Lactobacillus reuteri* and bifidobacteria. Lett. Appl. Microbiol..

[34] Kešnerová L., Emery O., Troilo M., Liberti J., Erkosar B., Engel P. (2020). Gut microbiota structure differs between honeybees in winter and summer. ISME J..

[35] Huang W.-F., Solter L.F. (2013). Comparative development and tissue tropism of *Nosema apis* and *Nosema ceranae*. J. Invertebr. Pathol..

[36] Forsgren E., Fries I. (2010). Comparative virulence of *Nosema ceranae* and *Nosema apis* in individual European honey bees. Vet. Parasitol..

[37] Li J., Qin H., Wu J., Sadd B., Wang X., Evans J., Peng W., Chen Y. (2012). The Prevalence of Parasites and Pathogens in Asian Honeybees *Apis cerana* in China. PLoS ONE.

[38] Schwarz R.S., Moran N.A., Evans J. (2016). Early gut colonizers shape parasite susceptibility and microbiota composition in honey bee workers. Proc. Natl. Acad. Sci. USA.

[39] Scharlaken B., De Graaf D.C., Goossens K., Brunain M., Peelman L.J., Jacobs F.J. (2008). Reference Gene Selection for Insect Expression Studies Using Quantitative Real-Time PCR: The Head of the Honeybee, *Apis mellifera*, After a Bacterial Challenge. J. Insect Sci..

[40] Denman S.E., McSweeney C. (2006). Development of a real-time PCR assay for monitoring anaerobic fungal and cellulolytic bacterial populations within the rumen. FEMS Microbiol. Ecol..

[41] Daisley B.A., Pitek A., Chmiel J.A., Al K., Chernyshova A.M., Faragalla K.M., Burton J., Thompson G.J., Reid G. (2020). Novel probiotic approach to counter *Paenibacillus larvae* infection in honey bees. ISME J..

[42] Vásquez A., Forsgren E., Fries I., Paxton R., Flaberg E., Szekely L., Olofsson T.C. (2012). Symbionts as Major Modulators of Insect Health: Lactic Acid Bacteria and Honeybees. PLoS ONE.

[43] Kaznowski A., Szymas B., Jazdzinska E., Kazimierczak M., Paetz H., Mokracka J. (2005). The effects of probiotic supplementation on the content of intestinal microflora and chemical composition of worker honey bees (*Apis mellifera*). J. Apic. Res..

[44] Maggi M., Negri P., Plischuk S., Szawarski N., De Piano F., De Feudis L., Eguaras M., Audisio C. (2013). Effects of the organic acids produced by a lactic acid bacterium in *Apis mellifera* colony development, *Nosema ceranae* control and fumagillin efficiency. Vet. Microbiol..

[45] Anderson K.E., Ricigliano V.A., Mott B.M., Copeland D.C., Floyd A.S., Maes P. (2018). The queen’s gut refines with age: Longevity phenotypes in a social insect model. Microbiome.

[46] Daisley B.A., Chmiel J.A., Pitek A.P., Thompson G.J., Reid G. (2020). Missing Microbes in Bees: How Systematic Depletion of Key Symbionts Erodes Immunity. Trends Microbiol..

[47] Wongchawalit J., Yamamoto T., Nakai H., Kim Y.-M., Sato N., Nishimoto M., Okuyama M., Mori H., Saji O., Chanchao C. (2006). Purification and Characterization of α-Glucosidase I from Japanese Honeybee (*Apis cerana japonica*) and Molecular Cloning of Its cDNA. Biosci. Biotechnol. Biochem..

[48] Dussaubat C., Brunet J.-L., Higes M., Colbourne J.K., Lopez J., Choi J.H., Martin-Hernández R., Botías C., Cousin M., McDonnell C. (2012). Gut Pathology and Responses to the Microsporidium *Nosema ceranae* in the Honey Bee *Apis mellifera*. PLoS ONE.

[49] Vidau C., Panek J., Texier C., Biron D.G., Belzunces L.P., Le Gall M., Broussard C., Delbac F., El Alaoui H. (2014). Differential proteomic analysis of midguts from *Nosema ceranae*-infected honeybees reveals manipulation of key host functions. J. Invertebr. Pathol..

[50] Huang W.-F., Solter L.F., Yau P., Imai B.S. (2013). *Nosema ceranae* Escapes Fumagillin Control in Honey Bees. PLoS Pathog..

[51] Fleming J.C., Schmehl D.R., Ellis J.D. (2015). Characterizing the Impact of Commercial Pollen Substitute Diets on the Level of *Nosema* spp. in Honey Bees (*Apis mellifera* L.). PLoS ONE.

[52] El Khoury S., Rousseau A., Lecoeur A., Cheaib B., Bouslama S., Mercier P.-L., Demey V., Castex M., Giovenazzo P., Derome N. (2018). Deleterious Interaction Between Honeybees (*Apis mellifera*) and its Microsporidian Intracellular Parasite Nosema ceranae Was Mitigated by Administrating Either Endogenous or Allochthonous Gut Microbiota Strains. Front. Ecol. Evol..

[53] Li J.H., Evans J., Li W.F., Zhao Y.Z., DeGrandi-Hoffman G., Huang S.K., Li Z.G., Hamilton M., Chen Y.P. (2017). New evidence showing that the destruction of gut bacteria by antibiotic treatment could increase the honey bee’s vulnerability to Nosema infection. PLoS ONE.

[54] Huang Q., Evans J.D. (2020). Targeting the honey bee gut parasite *Nosema ceranae* with siRNA positively affects gut bacteria. BMC Microbiol..

[55] Evans J.D., Spivak M. (2010). Socialized medicine: Individual and communal disease barriers in honey bees. J. Invertebr. Pathol..

[56] Page P., Lin Z., Buawangpong N., Zheng H., Hu F., Neumann P., Chantawannakul P., Dietemann V. (2016). Social apoptosis in honey bee superorganisms. Sci. Rep..

[57] Evans J.D., Aronstein K., Chen Y.P., Hetru C., Imler J.-L., Jiang H., Kanost M., Thompson G.J., Zou Z., Hultmark D. (2006). Immune pathways and defence mechanisms in honey bees *Apis mellifera*. Insect Mol. Biol..

[58] Tan S.-Q., Zhang K.-Q., Chen H.-X., Ge Y., Ji R., Shi W.-P. (2015). The mechanism for microsporidian parasite suppression of the hindgut bacteria of the migratory locust *Locusta migratoria manilensis*. Sci. Rep..

[59] Abraham N.M., Liu L., Jutras B.L., Yadav A.K., Narasimhan S., Gopalakrishnan V., Ansari J.M., Jefferson K.K., Cava F., Jacobs-Wagner C. (2017). Pathogen-mediated manipulation of arthropod microbiota to promote infection. Proc. Natl. Acad. Sci. USA.

[60] Burnham A.J. (2019). Scientific Advances in Controlling *Nosema ceranae* (Microsporidia) Infections in Honey Bees (*Apis mellifera*). Front. Vet. Sci..

[61] Klassen S., VanBlyderveen W., Eccles L., Kelly P., Borges D., Goodwin P., Petukhova T., Wang Q., Guzman-Novoa E. (2021). *Nosema ceranae* Infections in Honey Bees (*Apis mellifera*) Treated with Pre/Probiotics and Impacts on Colonies in the Field. Vet. Sci..

[62] Cilia G., Fratini F., Tafi E., Turchi B., Mancini S., Sagona S., Nanetti A., Cerri D., Felicioli A. (2020). Microbial Profile of the Ventriculum of Honey Bee (*Apis mellifera ligustica* Spinola, 1806) Fed with Veterinary Drugs, Dietary Supplements and Non-Protein Amino Acids. Vet. Sci..

[63] Nanetti A., Ugolini L., Cilia G., Pagnotta E., Malaguti L., Cardaio I., Matteo R., Lazzeri L. (2021). Seed Meals from *Brassica nigra* and *Eruca sativa* Control Artificial *Nosema ceranae* Infections in *Apis mellifera*. Microorganisms.

[64] Borges D., Guzman-Novoa E., Goodwin P. (2021). Effects of Prebiotics and Probiotics on Honey Bees (*Apis mellifera*) Infected with the Microsporidian Parasite *Nosema ceranae*. Microorganisms.

[65] Baffoni L., Gaggìa F., Alberoni D., Cabbri R., Nanetti A., Biavati B., Di Gioia D. (2016). Effect of dietary supplementation of Bifidobacterium and *Lactobacillus strains* in *Apis mellifera* L. against *Nosema ceranae*. Benef. Microbes.

[66] Tejerina M.R., Benítez-Ahrendts M.R., Audisio M.C. (2020). *Lactobacillus salivarius* A3iob Reduces the Incidence of Varroa destructor and *Nosema* spp. in Commercial Apiaries Located in the Northwest of Argentina. Probiotics Antimicrob. Proteins.

[67] Leonard S.P., Powell J.E., Perutka J., Geng P., Heckmann L.C., Horak R.D., Davies B.W., Ellington A.D., Barrick J.E., Moran N.A. (2020). Engineered symbionts activate honey bee immunity and limit pathogens. Science.

[68] Floyd A.S., Mott B.M., Maes P., Copeland D.C., McFrederick Q.S., Anderson K.E. (2020). Microbial Ecology of European Foul Brood Disease in the Honey Bee (*Apis mellifera*): Towards a Microbiome Understanding of Disease Susceptibility. Insects.

[69] Alonso-Salces R.M., Cugnata N.M., Guaspari E., Pellegrini M.C., Aubone I., De Piano F.G., Antúnez K., Fuselli S.R. (2017). Natural strategies for the control of *Paenibacillus larvae*, the causative agent of American foulbrood in honey bees: A review. Apidologie.

[70] Dosch C., Manigk A., Streicher T., Tehel A., Paxton R., Tragust S. (2021). The Gut Microbiota Can Provide Viral Tolerance in the Honey Bee. Microorganisms.

