# Supplementary material for: Vairimorpha (Nosema) ceranae Infection Alters Honey Bee Microbiota Composition and Sustains the Survival of Adult Honey Bees
Source: Biology (Basel). 2021 Sep 13;10(9):905. doi: 10.3390/biology10090905 (PMC8464679; doi:10.3390/biology10090905)
Supplement: Supplementary file 1 [file biology-10-00905-s001.zip › biology-1310107-supplementary.pdf]

Table S1. Primers used in the qPCR

Primers	Sequences	Concentration[1]	Temperature[2]	References
Bifido-F	CAAGCGAGAGTGAGTGTACC	200pM	57°C	(Li et al 2012)
Bifido-R	GCCGATCCACCGTTAAGC			
Lacto-F	TAACGCATTAAGCACTCC	400pM	57°C	
Lacto-R	GCTGGCAACTAATAATAAGG			
S. alvi-F	CTTAGAGATAGGAGAGTG	200pM	58°C	(Schwarz et al 2016)
S. alvi-R	TAATGATGGCAACTAATGACAA			
G. api-F	GTATCTAATAGGTGCATCAATT	200pM	58°C	
G. api-R	TCCTCTACAATACTCTAGTT			
1114F	CGGCAACGAGCGCAACCC	150 pM	61°C	(Denman and McSweeney 2006)
1275R	CCATTGTAGCACGTGTGTAGCC			
Actin-F	TGCCAACACTGTCCTTTCTG	200 pM	59°C	(Scharlaken et al 2008)
Actin-R	AGAATTGACCCACCAATCCA			

[1] The final concentration used in the qPCRs

[2] The second (annealing/extension) temperatures used in the 2-step qPCRs

References

Denman SE, McSweeney CS (2006). Development of a real-time PCR assay for monitoring anaerobic fungal and cellulolytic bacterial populations within the rumen. *FEMS Microbiology Ecology* **58:** 572-582.

Li J, Qin H, Wu J, Sadd BM, Wang X, Evans JD *et al* (2012). The prevalence of parasites and pathogens in Asian honeybees Apis cerana in China. *PloS One* **7:** e47955-e47955.

Scharlaken B, de Graaf DC, Goossens K, Brunain M, Peelman LJ, Jacobs FJ (2008). Reference Gene Selection for Insect Expression Studies Using Quantitative Real-Time PCR: The Head of the Honeybee, Apis mellifera, After a Bacterial Challenge. *J Insect Sci* **8:** 33.

Schwarz RS, Moran NA, Evans JD (2016). Early gut colonizers shape parasite susceptibility and microbiota composition in honey bee workers. *Proceedings of the National Academy of Sciences* **113:** 9345-9350.

Table S2. ΔCq values used in Figures 4 and 5. Sampler numbers were listed in parentheses.

		**Hindgut (Fig.4)**[1]		Hindgut (Fig. 5A)[2]		Feces (Fig. 5B)[2]	
		Un-infected	Infected	Un-infected	Infected	Un-infected	Infected
Bifidobacterium	Sucrose	2.93±0.35 (52)	0.08±0.27 (49)	6.26±0.41 (43)	4.68±0.21 (47)	6.44±0.52 (36)	4.27±0.30 (47)
	IMO	1.78±0.46 (54)	0.06±0.28 (50)	5.33±0.36 (52)	4.57±0.27 (47)	5.58±0.43 (37)	4.30±0.30 (43)
Lactobacillus	Sucrose	-3.53±0.19 (52)	-4.81±0.23 (49)	-0.53±0.18 (43)	-0.13±0.17 (47)	1.52±0.32 (36)	1.87±0.17 (47)
	IMO	-3.90±0.31 (54)	-4.23±0.27 (50)	-0.48±0.20 (52)	0.21±0.24 (47)	2.55±0.35 (38)	1.79±0.26 (43)
Snodgrassella	Sucrose	0.68±0.65 (41)	-3.95±0.26 (49)	3.42±0.62 (34)	1.04±0.26 (47)	5.34±1.01 (26)	4.54±0.37 (47)
	IMO	-1.80±0.58 (49)	-3.09±0.45 (52)	2.05±0.45 (48)	1.44±0.49 (48)	6.77±0.66 (23)	4.24±0.40 (43)
Gilliamella	Sucrose	-2.21±0.90 (17)	-3.89±0.29 (53)	1.51±0.72 (13)	0.89±0.27 (50)	3.10±1.12 (6)	1.71±0.32 (49)
	IMO	-1.36±0.55 (28)	-3.88±0.35 (53)	2.43±0.45 (28)	0.80±0.27 (50)	4.47±0.49 (11)	1.54±0.49 (46)

[1] The values were normalized using the honey bee beta-actin gene.
[2] The values were normalized using the universal bacteria results.
